# Evaluation of Caco-2 and human intestinal epithelial cells as *in vitro* models of colonic and small intestinal integrity

**DOI:** 10.1016/j.bbrep.2022.101314

**Published:** 2022-07-18

**Authors:** Silvia Lopez-Escalera, Anja Wellejus

**Affiliations:** a Human Health Research, Scientific Affairs, Chr. Hansen A/S, Bøge Alle 10-12, DK-2970, Hørsholm, Denmark; b Friedrich-Schiller Universität Jena, Fakultät für Biowissenschaften, Bachstraβe 18K, 07743, Jena, Germany

**Keywords:** Tight junction, Transepithelial electrical resistance, Caco-2, HIEC, Gut barrier function, Probiotics

## Abstract

Although the colonic cell line Caco-2 is widely used as a model of the small intestinal barrier function, it has limitations such as overestimated transepithelial electrical resistance (TEER) compared to *in vivo* conditions. Therefore, we investigated Human Intestinal Epithelial Cells (HIECs) as an alternative *in vitro* model*.*

We explored whether cell seeding number of HIEC-6, and the number of incubation days for HIEC and Caco-2 cells had an impact on TEER, and tight junction expression was examined for both cell lines via immunofluorescence in the presence and absence of probiotic bacteria.

We observed no significant difference in TEER readings for either cell lines when cultured for different days. Further, the HIEC TEER readings did not change with increased seeding number and were not significantly different from a control with no cells. HIECs expressed Claudin-1 and Zonula Occludens-1 but not Occludin. Caco-2 co-culture with probiotic bacteria demonstrated a significant increase in TEER, particularly for the lactobacillus strains, whereas HIEC TEER did not respond to bacterial co-incubation.

Our study shows that although HIECs express certain TJ proteins, a significant TEER was not observed, likely due to the embryonic origin of the cells, which limits the application of this cell line as a suitable model for small intestinal barrier function.

## Highlights

• Human embryonic intestinal epithelial cells cannot form a significant barrier.
• Contrary to Caco-2 cells HIECs do not express the tight junction protein occludin.
• Probiotic bacteria induce a tight barrier in Caco-2 cells but not in HIECs.

## 1 Introduction

The small intestinal epithelium has two major functions, namely, absorption of nutrients and forming a tight barrier against the potentially harmful components we are exposed to via food. The desire to understand the human intestinal epithelium in more detail has led scientists to mimic the small intestinal microenvironment via *in vitro* models that resemble small intestine physiology [1,2]. Commercially available cell lines derived from the gut, such as Caco-2, HT-29 and T84 cell are immortalized, which facilitates handling of cells under laboratory settings and turns these cell lines into powerful models of intestinal permeability [1,[3], [4], [5]]. These *in vitro* intestinal systems, such as the Caco-2 cell line, are widely accepted as models to study permeability, physiological processes, and disease mechanisms.

After growth of Caco-2 cells to confluency *in vitro,* the cells will begin to differentiate and phenotypically resemble enterocytes. Nonetheless, differences between Caco-2 cells and small intestinal epithelial cells are observed as Caco-2 cells lack specific transporter expression and metabolizing enzymes [1,6,7], which emphasizes how Caco-2 cells do not fully represent small intestinal physiology. Furthermore, the cell-cell tightness of the cell line has been reported to be significantly stronger compared to the cell-cell tightness of the small intestine *in vivo* [8]. This implies a decreased paracellular flux and lower absorption of the Caco-2 cell line epithelium compared to the small intestinal epithelium, *in vivo*. Also, adult-derived human intestinal epithelial cell (HIEC) monolayers were better at predicting absorption of certain test drugs compared to Caco-2 cells [4,9,10].

The importance of recapitulating major features of the human intestinal epithelium in the best possible way has challenged research groups to develop primary cell lines with characteristics that better resemble the small intestinal physiology compared to the immortalized cell lines. Beaulieu JF isolated and characterized small intestinal HIECs from a 17–19 week old human fetus, called HIEC-6 [7,11]. The HIEC-6 cell line has been thoroughly characterized and, besides the undifferentiated epithelial morphology, it also expresses crypt features and intestinal cell markers such as brush border enzymes and keratins [2,12]. Different research groups have studied this model for regulation of cell proliferation, mechanism of FAS-induced apoptosis, lipid metabolism [2], and drug absorption [9], however, to the best of our knowledge the epithelial barrier function of HIEC-6 cells has not been well characterized.

A widely used and well-established method to evaluate the integrity and tightness of epithelial monolayers is to measure transepithelial electrical resistance (TEER) across the cell layer. TEER has previously provided insights to the integrity of tight junctions (TJs) between neighboring cells [4,13]. This can be elegantly done via a cellZscope, which is an advanced automated system conducting repeated real-time TEER measurements.

TJs are dynamic structures that are located apically on the epithelial cells creating “kissing points” to a corresponding protein present on the neighboring cell. The TJ proteins represented in the small intestine, Occludin and Claudin, are bound to the cytoskeleton via scaffolding proteins such as Zonula occludens-1 (ZO-1) and this assembly plays a pivotal role in regulation of barrier function [[13], [14], [15], [16], [17]]. Based on the physiology and functional properties of the gut, these proteins can be localized. For instance, most the claudin family (CLDN 3, 4, 7 and 8) can be expressed in the large bowel whereas claudin 1 and 2 have been found in villus and crypt cells of small intestine [18,19]. Ideally, a combination of methods should be used to evaluate permeability and localize TJ expression when using *in vitro* models. Immunofluorescence is a technique often used to visualize junction markers intracellularly through fluorophores [20,21]. A mixed approach has already been employed on Caco-2 systems, offering a more complex answer to how the cells are affected by challenges and stressors [22,23].

Likewise, interactions between epithelial cell lines and probiotic bacteria have been extensively studied [[22], [23], [24]] By definition, probiotic bacteria are “live microorganisms which when administered in adequate amounts confer a health benefit on the host” [25]. In specific cases, probiotic bacteria have been shown to strengthen barrier integrity and increase gene expression of TJ-related structures [23,26].

Current knowledge around primary human small intestine cell models and their application for assessing barrier function is limited which encouraged us to investigate TEER and TJ expression in HIEC-6 cells as a potentially alternative model. Additionally, host-microbe interaction was studied by evaluating the junction strengthening effect of well-established probiotic bacteria.

## 2 Material and methods

### 2.1 Cell culture

HIEC-6 cells (ATCC-CRL 3266) were maintained on OptiMEM 1 Reduced Serum (Gibco 31985) and supplemented with 4% fetal bovine serum (FBS, Gibco 10270), 20 mM HEPES (1 M, Thermofisher CAT no. 15630080), 10 mM GlutaMAX (Gibco 35050), 10 ng/mL Epidermal Growth Factor (EGF, Sigma SRP3027) reconstructed with 5% Trehalose (Sigma T0167); Penicillin-Streptomycin solution (10000 units Penicillin G Sodium Salt and 10 mg/mL streptomycin sulfate) (Sigma, 69-57-8, 3810-74-0) hereafter referred as complete OptiMEM. Cells were grown in T175 cm^2^ plastic culture flasks (Thermo Scientific 15991) in humidified atmosphere 37 °C with 5% CO_2_. Cells used were passage number 5 to 15 for the assay.

The human colon adenocardinoma Caco-2 cell line (DSMZ ACC 169, Leibniz-Institut DSMZ-Deutsche Sammlung von Mikroorganismen und Zellkulturen GmbH, Braunschweig, Germany) was cultured in DMEM (Gibco™ 21885-025) and supplemented with 20% Fetal bovine serum (FBS Gibco™ 10270), 1% MEM non-essential amino acids (Biowest, X0557-100) and 1% Pen-Strep-Amp B (Biological Industries, 03-033-1B), hereafter referred to as complete DMEM. Cells were grown in T75cm^2^ culture flasks (ThermoScientific 174952) and kept in similar conditions as aforementioned HIEC-6. Cell passage numbers ranging between 9 and 20 were used for the experiments.

### 2.2 Bacteria culture

*Lacticaseibacillus rhamnosus* GG (formerly known as *Lactobacillus rhamnosus* GG, hereafter referenced by the use of the trademark LGG®)*, Bifidobacterium animalis* subsp*. lactis*, BB-12, hereafter referenced by the use of the trademark BB-12® and *Lacticaseibacillus paracasei* subsp. *paracasei* CRL 431 (formerly known as *Lactobacillus paracasei* subsp. *paracasei* CRL 431 and hereafter referenced by the use of the trademark L.CASEI 431®) were supplied by Chr. Hansen A/S, Denmark. *Lacticaseibacillus* strains were inoculated from frozen stock and grown overnight at 37 °C in Man-Rogosa-Sharpe (MRS) medium pH 6.5, whereas BB-12® was cultured anaerobically in anaerobic boxes with AnaeroGen™ pads (Thermo Scientific, AN0035A) at 37 °C in MRS supplemented with 0.05% cysteine hydrochloride monohydrate (CyHCl, CAS 7048-04). For TEER studies, 10-fold dilution series up to 1 × 10^−7^ were grown overnight at 37 °C.

After overnight incubation, bacterial cultures at late exponential/early stationary phase were selected based on their optical density at 600 nm wavelength (OD_600_). Bacterial cultures were centrifugated at 3500 g for 10 min at 19 °C, and the resulting pellets were washed in Hanks' Balanced Salt Solution (HBSS; Gibco, 14175) at 37 °C and re-suspended in the same media. Washing was repeated twice with a final resuspension in antibiotic-free complete OptiMEM or DMEM for HIEC-6 and Caco-2 cells, respectively. Subsequently, bacterial suspensions were adjusted to OD_600_ of 4.0.

### 2.3 Transepithelial electrical resistance (TEER)

Three TEER experiments were performed to characterize barrier function and expression of TJ proteins in HIEC-6 and Caco-2 cells and to investigate the interaction with lactobacilli and bifidobacteria.

In the first experiment, the aim was to investigate if the TEER read outs varied following different number of days in culture. Briefly, upon HIEC-6 and Caco-2 cells reached 80% confluency were treated with TrypLE Express Enzyme (Gibco, 12604) and seeded apically at 1 × 10^4^ cells/well and 1 × 10^5^ cells/well onto 12-well, 12 mm Transwell® with 0.4 μm pore polyester membrane insert (Corning; 3460) respectively; as previous described by *Tatenaka* et al. *2014*. Growth media was renewed every second day with complete OptiMEM and DMEM, correspondingly. A TEER assay was performed after 6, 8, 10 and 12 days in culture for the HIEC-6 cells whereas Caco-2 were grown for 15, 18, 21 and 24 days in culture. The day prior to the TEER experiment, transwell inserts were transferred to the CellZscope2 (Nanoanalytics, Munster, Germany) and the medium was changed to antibiotic-free medium (ABx) by adding 800 μL to the apical and 1.5 mL to the basolateral compartment, respectively. The CellZscope2 was maintained in a humidified atmosphere at 37 °C with 5% CO_2_. The resistance was measured hourly by automated data collection overnight for 18 h.

The aim of the second experiment was to investigate whether the number of cells used for seeding would impact the TEER for HIEC-6 cells. The HIEC-6 cells were seeded on transwells at cell densities ranging from 1 × 10^2^ to 1 × 10^7^ cells/well, and cell handling, maintenance and the TEER run conditions were similar as previously mentioned. Resistance was measured after 12 days and 15 days in culture. Further, the cells were kept in culture for 3 additional days followed by immunofluorescence staining of tight junctions as described below.

In the third experiment, HIEC-6 and Caco-2 cells were co-incubated with lactobacilli and a *Bifidobacterium* to explore the impact of probiotic bacteria on TEER and TJ protein expression. As described previously, HIEC-6 cells were maintained in complete OptiMEM and grown for 10 days on transwells, whereas Caco-2 cells were grown for 21 days in DMEM on transwells. Inserts were transferred to the CellZscope2 the day before the experiment, and cell culture media was changed to ABx. The CellZscope2 was kept in a humidified atmosphere at 37 °C with 5% CO_2_ overnight before the experiment started to allow for determination of baseline TEER in each well and served as a baseline and a quality control of a stable electrical resistance. After hourly overnight readings, the CellZscope2 was paused and 100 μL of apical medium was replaced with 100 μL of OD adjusted bacterial cell suspension or ABx alone. The final OD_600_ of the bacterial suspensions was 0.5. Each condition was measured in triplicate. The CellZscope2 was transferred back to the incubator and readings were resumed for a total of 18 h. Changes in TEER during bacterial stimulation were calculated relative to the latest value recorded immediately prior to the stimulation (baseline measurement were set to 100%). Area under curve (AUC) was calculated for each condition.

### 2.4 Immunofluorescence of tight junctions (occludin, Zonula occludens-1 and Claudin-1)

Following TEER measurements of experiment 2 and 3, the HIEC-6 and Caco-2 transwells were transferred to 12-well plates containing Phosphate Buffer Solution (PBS, Sigma 806552). Each transwell was carefully washed once with PBS and the cell membranes were fixed with 4% formaldehyde (VWR 1.004.968.350) for 6 min at room temperature (RT) and washed three times in PBS. Treated membranes were then removed from the transwells using a scalpel and transferred into a 48 well-plate (Costar 3548) with PBS. Subsequently, cell permeabilization was ensured by treating the membranes with 0.2% Triton X-100 (Sigma A4503) for 5 min at RT followed by a washing cycle with PBS. Blocking was done by treating the membranes for 1 h with 2% Bovine Serum Albumin (BSA, Sigma A4503). Primary antibodies were diluted in BSA as described in Table 1 and the treated membranes were incubated overnight at 4 °C on a platform rocker (14 inclination with 5 s intervals) (Mimetas, Organo-flow L-191000059). The membranes were washed three times with 0.25% BSA and 0.1% Triton X-100 in PBS (washing buffer) and were then incubated for 2 h in the dark at RT with secondary antibodies (diluted in washing buffer according to descriptions in Table 1). Following incubation with the antibodies, the slides were washed twice with PBS and labelled with nuclei staining; 4′,6-diamidino-2-phenylindole (DAPI, Life Technologies D1306), by incubation for 5 min, protected from light. The slides were washed three times with deionized water. Upon immunofluorescence protocol completion, membranes were transferred onto 18 mm slides (VWR® European 631–1550), mounted with fluorescence mounting medium (Agilent S3023) and coverslips (Karl Hecht GmbH, 41000109). Staining was visualized via a fluorescence microscope EVOS™ FL Auto 2D (Invitrogen, AMAFD2000) using 10x, 20x and 40× magnification.

**Table 1:** Primary and secondary antibodies.

Antibody/protein	Function	Dilution	Product ID
Occludin	Tight junction marker Primary antibody	1:100	Thermofisher 61-7300
Zonula Occludens-1	Tight junction marker Primary antibody	1:200	AH Diagnostics Sc-133256
Claudin-1 (A-9)	Barrier function & TJ Primary antibody	1:100	Santa Cruz Biotechnology Sc-166338
Goat anti-mouse, Alexa flour 488	Secondary antibody	1:1000	Life Technologies A-11001
Donkey anti-rabbit, Alexa flour 555	Secondary antibody	1:1000	ThermoScientific A31572

## 3 Statistics

The impact of days after seeding and seeding number were tested statistically by using the one-way ANOVA test on the 18 h timepoints for Caco-2 and HIEC-6 cells, separately. In the co-incubation experiment, cell cultures exposed to bacterial suspension were compared to control wells via the one-way ANOVA test. Statistics were calculated using the software GraphPad Prism (v. 9.3.1 GraphPad Software, LLC.)

## 4 Results

### 4.1 Transepithelial electrical resistance (TEER)

**Fig. 1:**
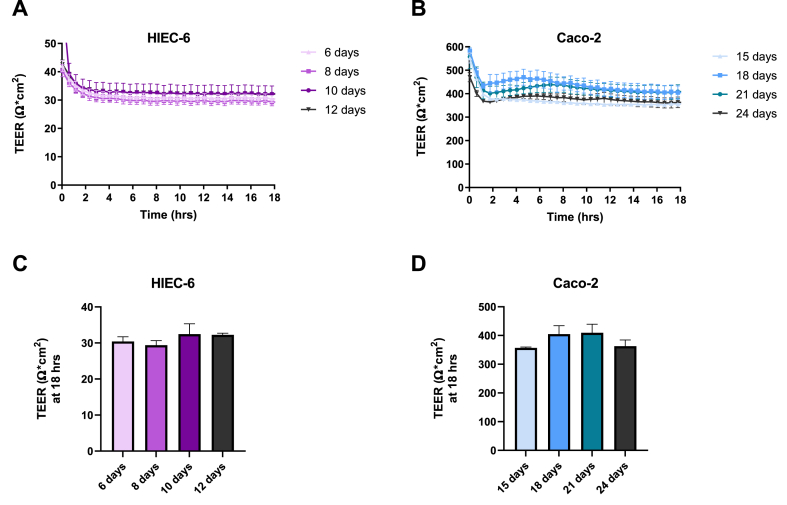
Baseline TEER measurements after different incubation days in HIEC-6 (A) and Caco-2 (B) cell monolayers. Average baseline readings after 18 h in the CellZscope for HIEC-6 and Caco-2 cells are shown in C and D, respectively. Data represent mean ± SD (n = 3).

In order to determine the best incubation period to achieve a tight cellular monolayer, HIEC-6 cells were incubated for 6, 8, 10, 12 days on transwells at a seeding density of 10^4^ cells/well whereas Caco-2 cells were seeded at 10^5^ cell/well grown for 15, 18, 21 and 24 days on transwells after seeding. As shown in Fig. 1, TEER measurements were not significantly affected by different incubation times in either of the two cell lines. Although not significantly different, Caco-2 cells showed higher TEER mean values after 18 days and 21 days of incubation compared to 15 days and 24 days in culture. Initial peaks indicate cells adapting to the CellZscope2 and incubator's atmosphere during the first measurements. The low TEER measurements of the HIEC-6 monolayer led us to further investigate whether seeding densities could affect the TEER readings.

**Fig. 2:**
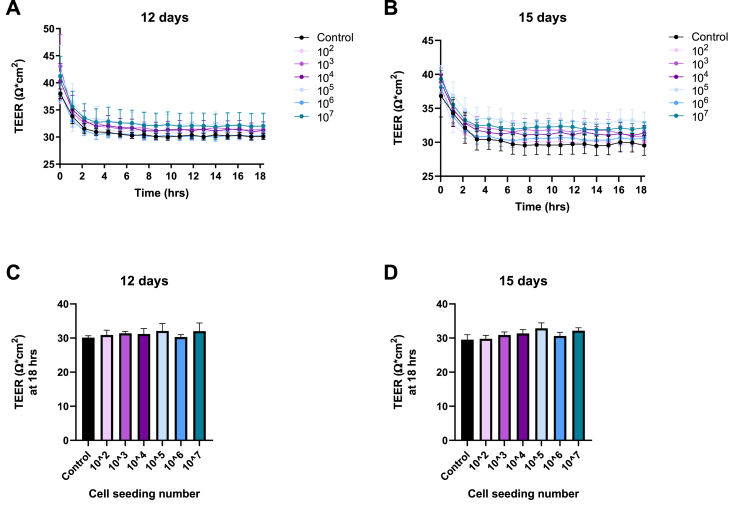
Baseline TEER measurements of HIEC-6 cell monolayers after seeding with increasing number of cells (from 10^2^ to 10^7^ cells/well) at incubation day 12 (A) and day 15 (B). Controls are cell-free Transwell insert with OptiMEM media only. Baseline readings after 18 h in the CellZscope after 12 days(C) and 15 days (D) of growth. Data shown represent the mean ± SD (n = 3).

Since few studies have been conducted using HIEC-6 cells, the incubation period was extended until day 15 and two TEER measurements of 18 h each were performed at incubation day 12 and 15. In order to standardize HIEC-6 protocol and determine best seeding concentration to obtain a tight monolayer, we performed a dose-dependent seeding experiment. Cells were seeded on transwells using 1 × 10^2^ to 1 × 10^7^ cells/well. Interestingly, none of the seeding densities increased TEER values regardless of the number of days in culture (Fig. 2). Even though the higher densities of seeded cells ranging from 1 × 10^4^ to 1 × 10^7^ reached confluency, TEER values remained comparable to control conditions (transwells without cells) and to the less confluent monolayers (Fig. 2). No significant differences in TEER readings were found between cell densities or incubation days compared to controls. TEER mean values were between 32.24 ± 2.8 Ω x cm^2^ and 31.5 ± 5.01 Ω x cm^2^ on day 12 and 15 respectively, which were similar to TEER measurements from the initial experiment (Fig. 1).

### 4.2 Tight junction identification

The aim was to further understand the lack of increase of TEER with increased seeding cell number and prolonged incubation. Therefore, the tight junction expression pattern was evaluated according to cell seeding density after 18 days in cell culture.

**Fig. 3:**
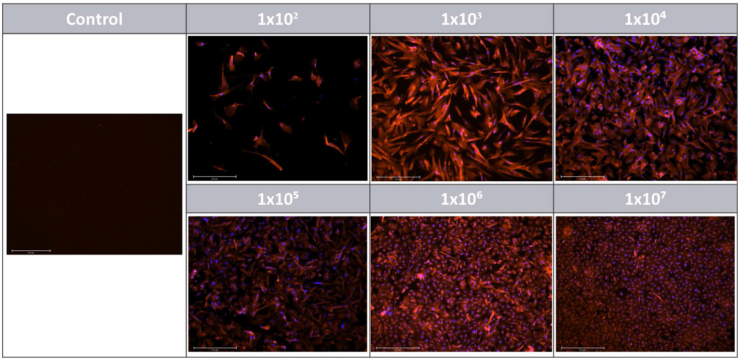
Zonula Occludens-1 (red) and nuclei (blue) identification by immunofluorescence at different HIEC-6 cell seeding densities after 18 days of incubation. Magnification 10×. Scale bar is 275 μm. Control is cell-free Transwell insert with OptiMEM media only and the negative control (secondary antibody alone) did not exhibit any fluorescence (data not shown). (For interpretation of the references to colour in this figure legend, the reader is referred to the Web version of this article.)

Occludin and Zonula Occludens-1 (ZO-1) are extensively investigated junctional complexes [15,[27], [28], [29]], therefore these proteins were selected in order to be localized within HIEC-6 cells. ZO-1 was expressed along the cell membrane as seen in Fig. 3 regardless of the cell density. Occludin, on the other hand, was not detected in any of the membranes (data not shown).

**Fig. 4:**
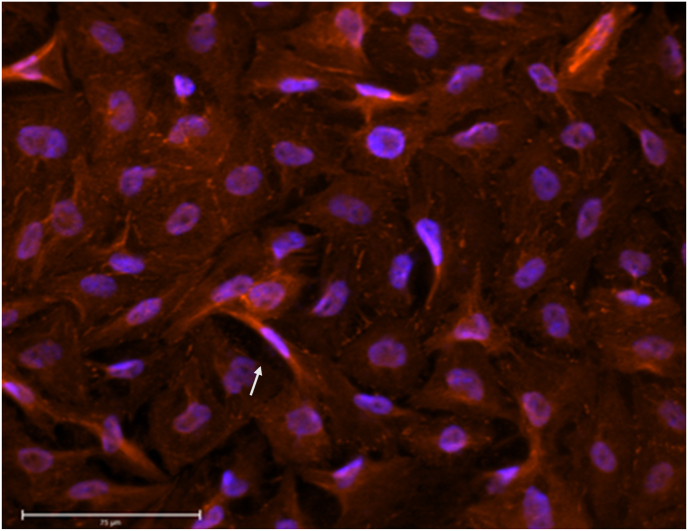
18-days-old HIEC-6 monolayer at 10^7^ cell concentration. White arrow indicate inter-cellular gap. Magnification 40×. Scale bar 75 μm.

Moreover, based on the bioimaging results, variations in the morphological and structural features of HIEC-6 cells were observed. At the highest density (1 × 10^7^), cell silhouettes became geometrical rather than preserving their elegant, elongated structure which was observed at lower densities (≤10^5^) (Fig. 3), nonetheless, TJ localization remained unaffected and was evenly distributed. Despite cells reaching confluency after 18 incubation days, cellular gaps were still present in the monolayer (indicated by arrow in Fig. 4).

### 4.3 Claudin-1 expression

Because of the limited capacity of HIEC-6 cells to express the Occludin protein, we aimed to identify a transmembrane junction strand. Claudins have been demonstrated to constitute part of TJ [15] and multiple family members have been identified; i.e, claudin-1/2/3/4/7, however, in this study only Claudin-1 was examined.

**Fig. 5:**
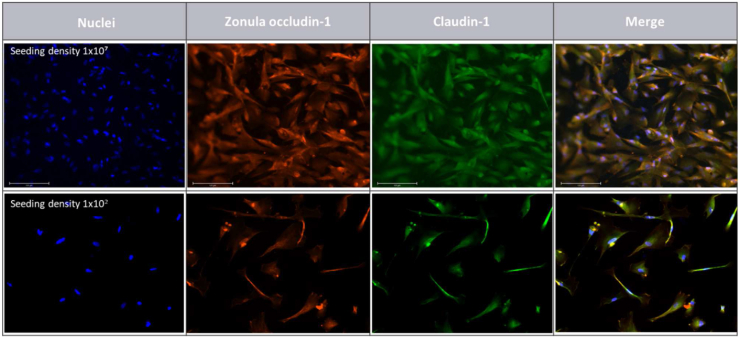
Immunoflourescence staining of HIEC-6 cells for Zonula occludin-1, Claudin-1 and nuclei when seeded in high density; 10^7^ cells/well (top row) and low density; 10^2^ cells/well (bottom row). Magnification 20×. Scale bar 125 μm.

As observed in Fig. 5, Claudin-1 was uniformly identified in HIEC-6 cells at both seeding densities tested (1 × 10^2^ and 1 × 10^7^). The immunofluorescence staining was an appreciable strand along the cells, indicating that this protein is expressed within the cytoplasm.

### 4.4 Bacterial co-incubation

**Fig. 6:**
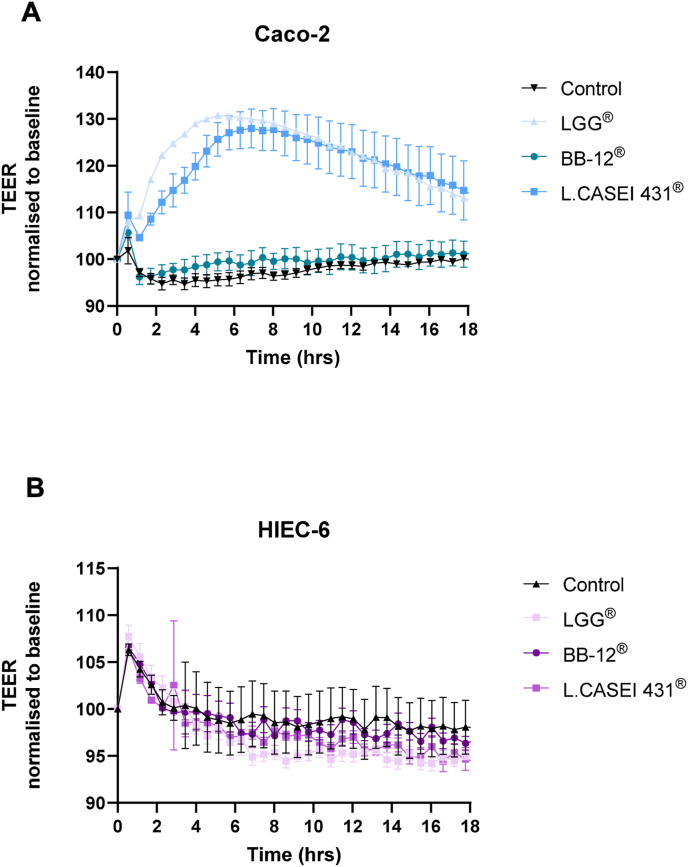
TEER readings normalized to baseline reading immediately before adding bacteria; LGG®; BB-12®; L.CASEI 431® and control (media only) for A) Caco-2 cells and B) HIEC-6 cells.

Caco-2 and HIEC response to bacterial co-incubation was also explored. Three different bacterial strains all representing industrially produced probiotic strains and representing different species were used to explore TEER response and TJ expression. Fig. 6 shows LGG®, L.CASEI 431® and BB-12® co-incubated with Caco-2 and HIEC-6, respectively. LGG® and L.CASEI 431® both induced high TEER readings compared to baseline, whereas BB-12® only showed limited effect on TEER when co-incubated with Caco-2 cells. In HIEC cells, no response to either of the bacteria was observed, this is consistent with the previous finding that showed confluent cells that were not tight enough to generate a significant TEER reading.

**Fig. 7:**
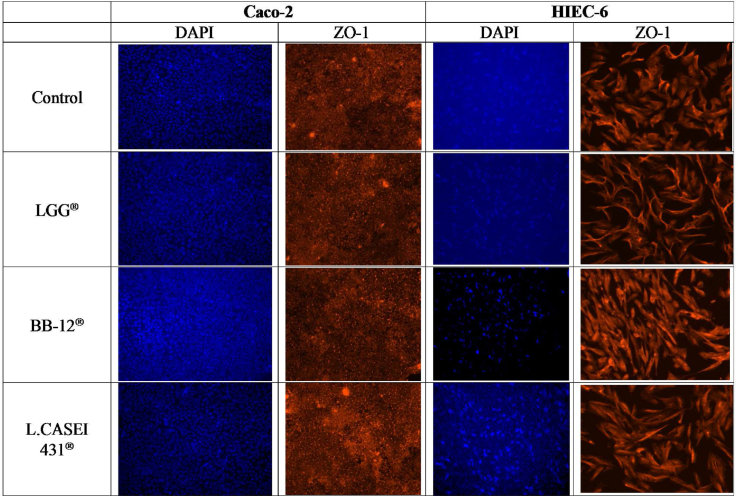
Immunoflourescence staining of Occludin, Zonula Occludens-1 and nuclei for Caco-2 cells, and Zonula Occludens-1 and nuclei for HIEC-6 cells after co-incubation with LGG®; BB-12®; L.CASEI 431®. Magnification 20×. Scale bar 125 μm.

In Fig. 7 staining of the HIEC-6 and Caco-2 cell membranes are shown after bacterial co-incubation. In the Caco-2 cells, ZO-1 showed a consistent spiderweb-like staining pattern on the cell periphery that did not change upon bacterial exposure. In contrast, ZO-1 staining in HIEC-6 appeared different according to which bacterium was used for co-incubation compared to control, in which labeling appears uniformly within the cytoplasm. Upon LGG® exposure, ZO-1 staining was localized intercellularly especially close to the cell periphery. Interestingly, HIEC-6 showed a change in morphology to an elongated structure and more intense homogenous fluorescence after BB-12® co-incubation compared to control. No changes were observed on HIEC-6 co-cultured with L. CASEI 431® compared to control.

## 5 Discussion

Previous investigation of HIEC monolayers indicate superior drug absorption prediction [30] and better physiological representation of small intestinal conditions compared to Caco-2 cells [9]. In this study, we evaluated HIEC-6 cells as a model of intestinal barrier function. Previously, Perreault and Beaulieu [31] showed ZO-1 expression and desmosome protein ZK-31 in this cell line, yet its permeability and transepithelial barrier capacity has not been fully investigated. Our barrier integrity experiments did not indicate any increased TEER when using HIEC-6 cells as monolayers compared to control (no cells), regardless of high cell density and a broad range of incubation days. Our TEER readings for the HIEC cells were similar to the TEER readings for the control with no cells, strongly indicating that measurements correspond to resistance across the transwells and not across the cell layer itself. In the publication by Takenaka et al. [9]*.*, the HIEC cells were also seeded at a density of 1 × 10^5^ cells/well on 24-well fibrillar collagen–coated inserts and at a density of 6.3 × 10^4^ cells/well on 12-well noncoated membrane inserts, and TEER was measured daily. After day 5, TEER readings reached a plateau at approximately 100 Ω*cm^2^ that lasted at least until day 11. We were not able to reproduce the tight monolayer formed by the HIEC cells and the increase of TEER as previously found [9]; even though we did prolong growth and differentiation phase on the membranes and increase the number of cells seeded. In support of this, probiotic bacteria were not able to induce an increase in TEER readings. *In vivo*, the human small intestine has lower TEER compared to the colon whereas *ex vivo* measurements using the Ussing chamber showed values of (45 ± 21 Ω x cm^2^), (34 ± 12 Ω x cm^2^) and (37 ± 4.36 Ω x cm^2^) for duodenum, jejunum and ileum, respectively, however not all of the samples were derived from healthy subjects [32]. This suggests a less tight epithelial layer allowing for increased passage of macromolecules. In the HIEC cells of human embryonic origin, the missing TEER development across the cell membrane and the notorious holes observed via immunofluorescence suggest an immature epithelial cell line. This is in line with the impaired gut barrier function observed during early life in many mammalian species, which allows for passive transmission of immunoglobulins from the mother's milk [33], for example. Interestingly, the HIEC cells seemed to differentiate and showed an epithelial-like phenotype after 18 days in culture seeded at a density of 1 × 10^7^ (Fig. 5) compared to cells seeded at a lower density, indicating that cell-cell contact may lead to physiological cell changes.

To better understand HIEC-6 transepithelial resistance, we used an immunofluorescence technique on TJ proteins. TJ features have been identified on Caco-2 cells [34] and Occludin and ZO-1 are well established junction proteins that have been localized on these colonic cells [35,36]. In contrast, little is known about TJ expression in HIEC cells. Our fluorescence results indicate that this cell line does not express Occludin, which could explain the lack of TEER since this protein had been thought of as essential part for anchoring adjacent cells [16,17]. However, studies on Occludin^−/−^ mice showed that its absence did not impair barrier function [37,38] whereas it did cause alterations on paracellular flux mainly in gastric mucosa [39]. In this study, HIECs expressed ZO-1, which is in line with previous findings in which ZO-1 was expressed in differentiated epithelial cells from Occludin-deficient embryonic stem cells, and thus ZO-1 seems essential to form TJ in embryonic tissue [15,27]. ZO-1 is responsible for anchoring Occludin and Claudin to Actin in the cytoskeleton [16] through the PDZ-domain, and this finding led to the identification of the Claudin family as one of the backbones of the TJ networks [27,37,40].

Due to the absence of Occludin expression in the HIEC cells and the desire to better understand the TJ composition in these cells, we investigated Claudin-1 protein expression in HIEC-6. TJ assembly begins basolaterally with ZO-1 binding adherens junctions henceforth recruiting primodial junctions (α and β catenins) as junctions continue to mature. ZO-1 then migrates upwards until maturation and Occludin interacts with junctional adhesion molecules-A, culminating in accumulation of Claudin apically [15,37]. Although fetal small intestinal cells at 10–12 weeks of gestation have been reported to express most of the differentiated features observed in cells from adults [11]. HIEC cells represent cryptcells that are of a proliferative and undifferentiated nature [7]. In the current study, we substantiate these findings by underlining that these cells are not at a maturation stage in which they can generate a TEER across the cell layer and a tight paracellular monolayer.

In contrast, Caco-2 monolayers, originating from colonic tissue, are widely used as a model of intestinal integrity and seem to generate higher TEER readings, although large interexperimental variation exist due to the different methodologies used, such as chopsticks, CellZscope, MiilCell-ERS system and EVOM Endohm [4,9,32,41].

Interestingly, previous studies have shown that barrier function may be significantly compromised or strengthened by co-incubation with bacteria, bacterial components or metabolites. Several pathogens have shown detrimental effects on barrier function by direct invasion of the epithelial cells and disruption of the TJs, as observed by *Salmonella thyphimurium* and enterotoxigenic *Escherichia coli* (ETEC) [42].

Probiotic bacteria, including lactobacilli and bifidobacteria have been acknowledged to affect barrier function via various mechanisms. For instance, the cell wall polysaccharide fraction of *B.breve* has shown anti-ulcer and anti-erosion effects in gastric models of acetic acid and ethanol-induced erosions [43]. Furthermore, *B.breve* has been associated with expression of Tad pili that stimulate colonic epithelial cell proliferation [44] that may support barrier function further. Additionally, secreted molecules including metabolites such as short chain fatty acids and indoles acting via the Aryl Hydrocarbon receptor [24,45] improve gut barrier function. The LGG® strain was reported to secrete peptides; p40 and p75, responsible for strengthening the barrier integrity and protect against H_2_O_2_-induced damage [23,46]. In this study, three probiotic bacteria were assessed as co-culture with Caco-2 cells, and all three showed increased TEER measurements compared to control, although to various degrees. LGG® was previously reported to induce increases in TEER when using fresh cultures [41], freeze dried product [47] and when present in a yogurt matrix [48]. On the other hand, BB-12® generated lower TEER readings compared to LGG® and L.CASEI 431®^,^ which has been previously reported [41]. This could be due to lower survival rate of BB-12® compared to the *Lactobacillus* strains that are generally more aerotolerant.

## 6 Conclusions

The data from this study suggest that despite HIECs being of non-immortalized gut epithelium origin, the cells do not form a confluent monolayer and therefore transepithelial electrical resistance cannot be measured across the cell layer regardless if the cells are stimulated with probiotic bacteria. The absence of Occludin in the HIECs supports the more immature profile compared to Caco-2 cells. In conclusion, HIECs do not seem suitable as a model for mimicking gut barrier function *in vitro*.

## Declarations of competing interest

Both authors Silvia Lopez-Escalera and Anja Wellejus are employed at Chr. Hansen A/S.
